# Comparing two-dimensional ultrasonography with three-dimensional ultrasonography and MRI for the levator ani defects grading

**DOI:** 10.1038/s41598-022-13427-3

**Published:** 2022-06-02

**Authors:** Yijia Luo, Honghong Pan, Linxin Yang, Ning Lin, Zhihua Fan, Weiji Chen

**Affiliations:** 1 Shengli Clinical Medical College of Fujian Medical University, Fuzhou, 350001 China; 2 Department of Ultrasound, Fujian Provincial Hospital, 134 Dongjie, Fuzhou, 350001 China; 3 Department of Urology, Fujian Provincial Hospital, Fuzhou, 350001 China

**Keywords:** Diagnosis, Health care, Magnetic resonance imaging, Three-dimensional imaging, Tomography, Ultrasonography, Urological manifestations, Reproductive signs and symptoms, Urogenital diseases

## Abstract

Levator ani defect (LAD) closely correlates with pelvic floor disorders (PFD). In general, LAD was graded by three-dimensional ultrasonography (3D-US) and MRI, which could be used hardly in some developing area. Our objective was to determine whether two-dimensional ultrasonography (2D-US), a method that is almost universally accessible, could be used to diagnose the LAD. 129 Chinese women with PFD were recruited for the LAD grading by 2D-US and 3D-US and MRI. LAD was classified into intact, partial and complete avulsions. The puborectalis attachment width (PAW) was measured by 2D-US and with the software on the three-dimensional MRI-based LAD models. The results were compared and analyzed using the weighted kappa and the Pearson’s coefficient. Of the 119 patients, 64 were diagnosed with LAD by 2D-US, 70 were identified by 3D-US while 68 were confirmed by MRI. The LAD grading of 2D-US showed good agreement with MRI (kappa = 0.78, 95% CI 0.71–0.86) and 3D-US (kappa = 0.77, 95% CI 0.70–0.84). In regard to the consensus of partial or complete avulsions, 2D-US showed excellent good agreement with MRI (kappa = 0.86, 95% CI 0.73–0.97), superior than 3D-US with MRI (kappa = 0.55, 95% CI 0.36–0.71). Additionally, iliococcygeus avulsions detected by MRI (n = 7) were accompanied by complete puborectalis avulsions. The averaged PAW was 10.42 ± 5.57 mm measured by 2D-US, which correlated well with the results measured by MRI (Pearson’s coefficient = 0.90). 2D-US, 3D-US and MRI showed the good agreement on LAD diagnosis. Compared with MRI and 3D-US, 2D-US was comparable in grading LAD, especially complete avulsions.

## Introduction

Levator ani muscle (LAM), a broad muscular sheet covering the outlet of the pelvis, plays a vital role in maintaining the pelvic functions and supporting pelvic organs^1^. The LAM complex has three major components, puborectalis, iliococcygeus, and pubococcygeus^2^. Delivery-related traumas are the most important causes of levator ani defects (LAD)^3^. Vaginal deliveries lead to the enlargement of the levator hiatus and the substantial stretch of the LAM, which may cause the LAD^1^.

It has been published that LAD closely correlate with pelvic floor disorders, especially pelvic organ prolapses (POP) and stress urinary incontinence (SUI). The LAD appears to double the risk of anterior and central compartment prolapse^4,5^. Moreover, the size of the defect has a direct correlation with prolapse symptoms^6^. It is significant to grade the LAD for the better treatment, the prediction of related symptoms and the further investigations of PFD mechanism.

The LAD can be graded by magnetic resonance imaging (MRI), two-dimensional ultrasonography (2D-US) and three-dimensional ultrasonography (3D-US). MRI is considered as the most reliable reference due to its intense soft tissue contrast and discriminatory capacity^7,8^, though there is no gold standard. However, MRI has not been widely adopted in urogynecological practice due to its cost and availability. 3D-US has been emerged as a more cost-efficient tool to diagnose LAD^9^. Neither MRI nor 3D-US is universally available as opposed to 2D-US. For 2D-US, the LAM could be observed by an oblique parasagittal translabial approach^10^. Most previous studies used 2-dimensional (2D) planes of 3D-US and MRI to describe the morphology of LAM, which was not sufficient for the description of LAM, a three-dimensional structure. Within the specific postprocessing software, 3-dimensional magnetic resonance models (3D-MR-model) can be reconstructed for 3-dimensional overview and valid parameters of LAM.

So far, no prior studies have compared 2D-US, 3D-US and MRI on the grading of LAD and evaluated the value of 2D-US on the diagnosis of LAD. Our objective was to determine whether 2D-US, an effective imaging method that is universally accessible, could be used to grade the size of the LAD compared with MRI and 3D-US.

## Results

From November 2019 to September 2021, 129 patients were recruited in this research. Ten patients were excluded due to the exclusive conditions. The LAD grading of 119 patients were assessed, equal to 238 attachments assessed. The general demographic characteristics of patients were presented in Table 1. 90.8% patients (108/119) had given birth vaginally. Presenting complaints were urinary stress incontinence (59/119, 49.6%), prolapse symptoms (74/119, 62.2%) and voiding dysfunctions (21/119, 17.6%).

**Table 1:** General demographics of women with pelvic floor disorders (N = 119).

Variables	Measurement
Age, y, (IQR)	64 (53, 75)
BMI, kg/m^2^	24.09 ± 3.13
Gravidity, n, (IQR)	4 (2, 5)
Parity, n, (IQR)	3 (2, 4)

Pelvic organ prolapse grade	Number (number/total %)
I	15 (12.6%)
II	31 (26.1%)
III	43 (36.1%)
IV	30 (25.2%)

*BMI* body mass index, *IQR* interquartile range.

**Figure 1:**
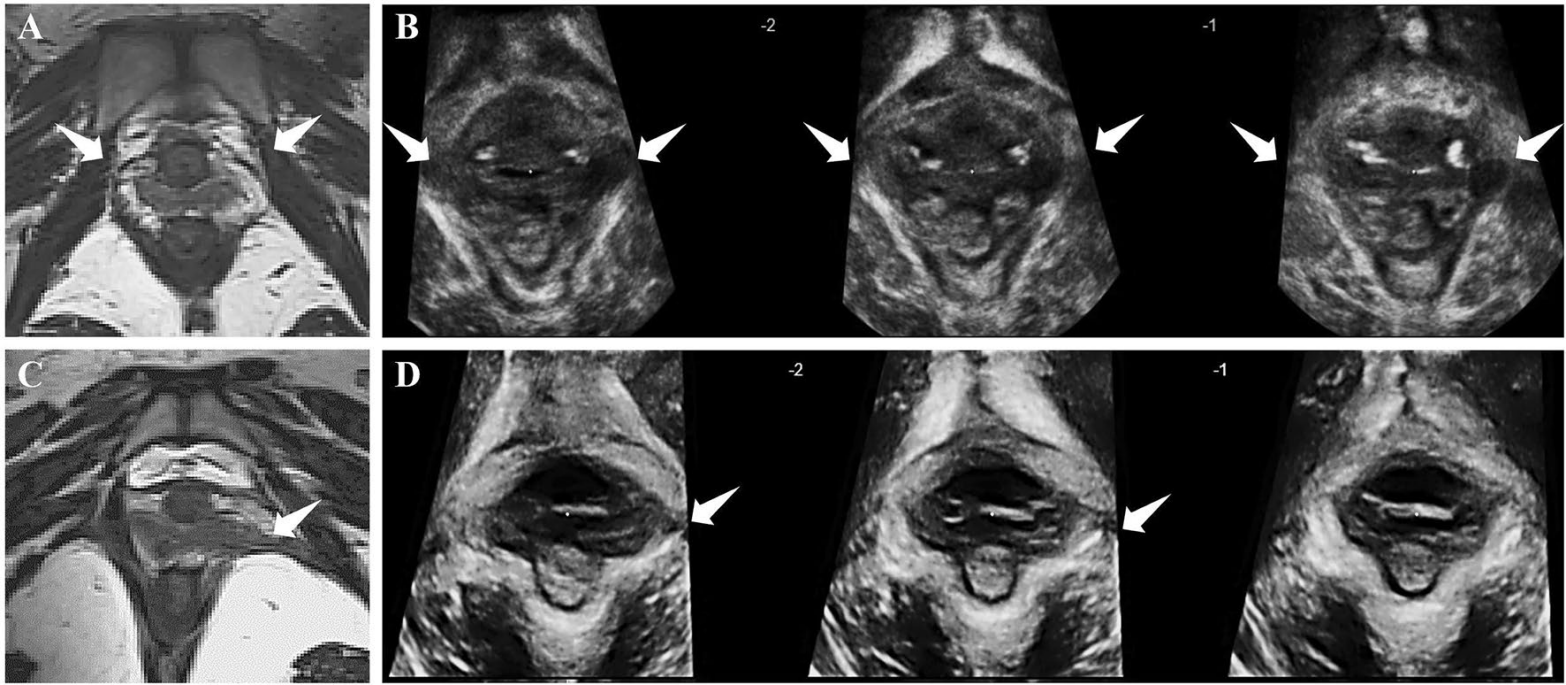
The magnetic resonance image showed the bilateral avulsion (**A**) and left avulsion (**C**). The tomographic ultrasound images showed complete avulsions at the both sides on the three central slices (**B**) and partial avulsion at left side on the three central slices (**D**). White arrows pointed the positions of the levator ani defect.

In the case of avulsions on 3D-US, the hypoechoic insertions appeared at the attachments of the puborectalis muscle at the pubic ramus. 49 patients had no visible avulsions. 48 patients had unilateral avulsions (Fig. 1D), while 22 had bilateral avulsions (Fig. 1B). 50 attachments showed partial avulsions and 42 presented complete avulsions.

In the case of avulsions on 2D-US, the attachments of puborectalis to the pelvis were replaced by the hypoechoic zones. 55 patients had no visible defects. 44 patients had unilateral avulsions. Meanwhile, 20 had bilateral avulsions. 55 attachments presented partial avulsions and 29 showed complete avulsions.

On MRI, 51 patients were diagnosed with no damage, while 41 had unilateral avulsions (Fig. 1C) and 27 had bilateral avulsions (Fig. 1A). 65 attachments were assessed as partial avulsions while 30 as complete avulsions. The iliococcygeus could be detected by MRI, similar to a butterfly. Seven patients were diagnosed with iliococcygeus avulsion accompanying with complete puborectalis avulsion at the same side.

Table 2 contained the comparison of the agreement on the LAD grading assessed by 3D-US, 2D-US and MRI. Both 2D-US and 3D-US agreed well with MRI in the extent of LAD (intact-partial-complete), with the kappa of 0.78 (95% Confidence interval [CI] 0.71–0.86) and 0.77 (95% CI 0.70–0.84) respectively. Further analysis showed that 2D-US presented the superior agreement (kappa = 0.86, 95%CI 0.73–0.97) than 3D-US (kappa = 0.55, 95% CI 0.36–0.71) in the diagnosis of partial or complete avulsions. However, 2D-US showed inferior agreement (kappa = 0.79, 95% CI 0.71–0.87) than 3D-US (kappa = 0.90, 95%CI 0.84–0.96) in the diagnosis of avulsions or not.

**Table 2:** Comparison of the agreement on the grading of levator ani defects between the MRI, three-dimensional ultrasonography and two-dimensional ultrasonography.

		MRI			MRI		Kappa	95% CI
		Intact	Partial	Complete				
2D-US	Intact	137	13	4	2D-US	Intact–Avulsion	0.79	0.71–0.87
	Partial	4	49	2		Intact–Partial–Complete	0.78	0.71–0.86
	Complete	2	3	24		Partial–Complete	0.86	0.73–0.97

		MRI			MRI		Kappa	95% CI
		Intact	Partial	Complete				
3D-US	Intact	139	5	2	3D-US	Intact–Avulsion	0.90	0.84–0.96
	Partial	1	45	4		Intact–Partial–Complete	0.77	0.70–0.84
	Complete	3	15	24		Partial–Complete	0.55	0.36–0.71

		3D-US			3D-US		Kappa	95% CI
		Intact	Partial	Complete				
2D-US	Intact	143	6	5	2D-US	Intact–Avulsion	0.87	0.81–0.93
	Partial	1	39	15		Intact–Partial–Complete	0.73	0.65–0.80
	Complete	2	5	22		Partial–Complete	0.49	0.29–0.68

*Avulsion* including partial avulsion and complete avulsion, *Intact* no avulsions, *Partial* partial avulsion, *Complete* complete avulsion, *MRI* magnetic resonance imaging, *2D-US* two-dimensional ultrasonography, *CI* confidence interval, *3D-US*, three-dimensional ultrasonography.

2D-US and 3D-US showed good agreement in the extent (intact-partial-complete) (kappa = 0.73, 95% CI 0.65–0.80) and the diagnosis of avulsion or not (kappa = 0.87, 95% CI 0.81–0.93). However, these two methods presented moderate agreement in the diagnosis of partial or complete avulsions (kappa = 0.49, 95% CI 0.29–0.68).

The puborectalis attachment width (PAW) of the intact attachments reached 10.42 ± 5.57 mm on 2D-US, while 11.01 ± 5.95 mm on 3D models. The agreement on the measurement of PAW showed excellent agreement with the Pearson’s coefficient of 0.90 (95% CI 0.83–0.97).

The kappa for the interobserver agreement of the LAD grading by the MRI was 0.83 (95% CI 0.76–0.89), which was defined as excellent agreement. The interobserver agreement between the two observers in LAD grading by 3D-US and 2D-US had the kappa of 0.79 (95% CI 0.73–0.85) and 0.77 (95% 0.71–0.83), respectively, which were defined as good agreement.

## Discussion

It has been proved that LAD closely correlates with PFD, especially anterior or central compartment prolapse^5,11^. It was also found that the size of LAD was associated with the severity of POP^4,6^. It is significant to grade the defect size for the better treatment and further researches of the PFD mechanism. The LAD has been usually detected and graded by 3D-US and MRI. This study was aimed to determine whether 2D-US, a more universally accessible method in hospital environments, could be used to grade the size of LAD compared with MRI and 3D-US.

3D-US is a non-invasive and widely clinically used method for LAD. It has remarkable advantages in real-time observation, easy-operation and efficiency^9,12^. However, 3D-US need the ultrasound diagnostic system equipped with volume transducer. MRI is recognized as the most reliable method for LAD grading due to its superior competence of soft tissue contrast^7^. However, MRI has not been widely adopted in clinical practice, the main reason being cost and availability. A majority of ultrasound diagnostic systems were equipped with abdominal curved array transducer. 2D-US could be much more widely clinically and economically used, even in some developing countries, if its repeatability and advantages in the LAD diagnosis has been authenticated.

According to the anatomy, the size of the levator avulsion was classified into no, partial and complete avulsions^2^. 2D-US, 3D-US and MRI used the different criteria to grade the size of LAD due to the different imaging principles and post-processing modes. In order to grade the extent of LAD, 3D-US used the number of the defect slices, MRI used the ratio of defect to no-defect slices, while 2D-US detected the discontinuity between the hyperechogenic muscle fiber and pelvic wall^4,9,10,13,14^. Previous studies had been proved that these different grading criteria on LAD was statistically objective, reliable and repeatable^7,10,11,15^. This study used the similar method as the previous studies^15–18^.

Several studies had compared MRI and 3D-US findings on LAD. Zhuang et al. and Notten et al. demonstrated that MRI and 3D-US correlated well in the grading of LAD^17,18^. However, their further analysis showed that the difference between the 3D-US and MRI was mainly in diagnosis of the partial or complete avulsions, which was in line with our findings. So far, no researches have compared 2D-US, 3D-US and MRI on LAD grading. This study compared these three imaging methods to prove the value of 2D-US. It was demonstrated that 3D-US was better in diagnosis of avulsions or not than 2D-US. Whereas 2D-US was better in diagnosing complete avulsions than 3D-US. On the grading of LAD (partial or complete), regarding MRI results as the standards, the diagnostic specificity was 75.0% and the diagnostic sensitivity of 3D-US reached 85.7%. If we used 3D-US to diagnose avulsions and then used 2D-US to confirm partial or complete avulsions, the diagnostic specificity promoted to 98.3% and the diagnostic sensitivity promoted to 92.3%. Therefore, 3D-US combined with 2D-US could better assess the grading of LAD. However, the iliococcygeus (the more cranial aspects of the LAM) was not invisible by translabial ultrasonography due to the poor resolution beyond 6 cm from perineum. The iliococcygeus avulsions could only be diagnosed by MRI. Moreover, this study also found that the iliococcygeus avulsions accompanied with the complete puborectalis avulsions at the same side, which has not been reported before.

Previous studies found that the difference between MRI and 3D-US mainly in diagnosing partial or complete avulsions^15,18^. So far, the reasons for the difference were remained unclear. This study proved that the three central slices on TUI (width of 7.5 mm) obtained by 3D-US could not cover the whole attachment with the width of 11.01 ± 5.95 mm, which resulted in the difference. In addition, the length of PAW measured by 2D-US agreed well with the measurement from 3D-MR-models, which illustrated that 2D-US could cover the whole attachment. Therefore, 2D-US had superior performance in grading partial or complete avulsions than 3D-US. Moreover, 2D-US could also observe the contraction and distention of the puborectalis attachments dynamically, which would contribute to the assessment and investigations of LAM contractility.

This study had several limits. First, the subject was not contained nulliparous women with intact LAM for comparison, which would be supplemented in the future works. Second, the PAW in contraction and distention was not recorded in this study, which contributed to assessing the functions of puborectalis.

In conclusion, compared with 3D-US and MRI, 2D-US is comparable in grading LAD, especially complete avulsions. In addition, 3D-US combined with 2D-US can more accurately assess the size of LAD.

## Methods

This study was conducted in Fujian Provincial Hospital from November 2019 to September 2021. Written informed consent was obtained from all participants.

This comparative research was undertaken in patients included from gynecological department, who were to undergo treatment for prolapse. 129 patients were interviewed about the symptoms of urinary or fecal incontinence using a standardized questionnaire^19^. They also underwent the prolapse assessment using the prolapse grading system of the International Continence Society^20^. Pelvic organ prolapse grading included five stages. Stage 0 assigned if no prolapse, stage I if greater than 1 cm above the hymen, stage II if 1 cm or less proximal or distal to the plane of the hymen, stage III if greater than 1 cm below the plane of the hymen, but protruding no farther than 2 cm less than the total vaginal length and stage IV if eversion of the lower genital tract was complete^20^. The exclusion criteria were: (1) history of metal implantation; (2) claustrophobia; (3) history of abdominal or pelvic surgery; (4) inability to understand the instructions in Mandarin. MRI was carried out after 1 to 5 days after ultrasound examinations. The grading of LAD was carried out on left or right ends of LAM separately.

### Three-dimensional ultrasonography

The 3D-US was performed on patients in the lithotomy position by a GE Voluson E8 system (GE Kretz Technik GmbH, Zipf, Austria) with a RAB 4–8 MHz volume transducer. On 3D-US, the puborectalis muscle originated from the pubic internal surface and then formed a V-shaped sling bypassing the anorectal junction. On the basis of Dietz’s scoring criteria, the minimal distance in the axial plane between the posterior pubic symphysis border (hypoechogenic area) and the central anterior aspect of puborectalis loop (hyperechogenic muscles) was identified^9^. The plane of minimal axial dimension was identified by this line. During the pelvic floor maximum contraction, tomographic ultrasound imaging (TUI) was then used to acquire the slices at 2.5 mm intervals, from 5 below to 12.5 mm above the plane of minimal dimension (eight slices, Slice − 2 to Slice 5). Complete avulsions were identified if all three central slices (Slice 0 to Slice 2) showed hypoechoic insertions (Fig. 1B), while partial avulsions were defined if any of the Slice 0 to Slice 5 presented abnormal except what was complete avulsions (Fig. 1D). The length of levator urethra gap (LUG) was the distance between the urethral mucosa and smooth muscle or on the most medial aspect of the muscle insertion^9^. In equivocal cases, the insertions were identified as avulsions if the LUG was greater than 25 mm^9^. The LUG was measured in three central slices and then averaged.

### Two-dimensional ultrasonography

**Figure 2:**
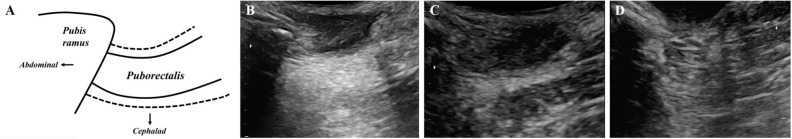
Parasagittal view of the puborectalis attached to the pubic ramus. (**A**) presented the schematic drawing of the principal structures of the attachment, which was seen in (**B**). (**B**) Showed the intact attachment of the puborectalis, with the hyperechogenic muscle fibers clearly visible. The black dotted line showed the measurement of the puborectalis attachment width (PAW). (**C**) Showed the partial avulsion of puborectalis at the attachment. (**D**) Presented the complete avulsion of puborectalis.

The 2D-US was undertaken by a GE Voluson E8 system (GE Kretz Technik GmbH, Zipf, Austria) with a 5–8 MHz convex array transducer. According to the anatomy, the puborectalis muscle attached to the pubis ramus (Fig. 2A). On 2D-US, the puborectalis represented the continuous hyperechogenic area attached to pubis (Fig. 2B). The main axis of transducer accorded with the fiber direction of the puborectalis by an oblique parasagittal approach. Starting from the mid-sagittal plane, the transducer was rotated by 10°–25° and tilted from the superolateral to the inferomedial direction^10^. The levator ani defects on 2D-US were classified into the intact, partial avulsion and complete avulsions. The intact levator ani muscle showed the hyperechogenic fibers attached to the pelvis without the discontinuity (hypoechogenic insertions) (Fig. 2B). Partial avulsions were identified if there was a discontinuity between the puborectalis hyperechogenic fibers and the pelvis but still remained hyperechogenic fibers attached to the pelvis^10^ (Fig. 2C). Complete avulsions were identified when no puborectalis fibers located on pelvic sidewalls (Fig. 2D).

The puborectalis attachment width (PAW) was measured as the length of origins from the pubic internal surface. We chose the most anterior section of puborectalis (0.5-1 cm) as the measuring range, as close as possible to the pubic internal surface (Fig. 2B). The PAW was measured three times and then averaged.

Two different observers (N. Lin and ZH. Fan) performed and assessed the 3D-US and 2D-US scans independently. They were blinded against patients’ demographics and previous examination findings. They were blinded to the measurements made by the other observer. At the start of the research, two observers had approximately 5 years of experience in performing and assessing the ultrasound scans (1000–1500) regarding pelvic floor. Furthermore, two observers confirmed the diagnostic standards before the study and participated in consensus meetings in which 22 3D-US images and 27 2D-US images were discussed.

### MRI examinations and three-dimensional modeling

The magnetic resonance images were obtained by a high-resolution axial 3 T scanner (Siemens, Erlangen, Germany) equipped with a 35 cm field of view. The standard imaging parameters were: T2-weighted turbo and fast spin echo sequence (TR, 1260 ms; TE 130 ms; slice thickness, 1.0 mm). According to Delancey’s scoring system, LAD was diagnosed if there was a discontinuity between the puborectalis and the pubic ramus (at least one 4 mm or two and more adjacent 2 mm sections in both the axial and coronal planes)^14^ (Fig. 1A,C). According to the size of avulsions, LAD was classified into the intact, partial avulsions and complete avulsions (no residual fibers)^14,21^. The iliococcygeus originated from the arcus tendinous levator ani, which overlies the obturator internus. The iliococcygeus avulsions were diagnosed if there was a discontinuity between the iliococcygeus and the obturator internus.

Each MRI scan was assessed offline by two researchers (YL. Xu and XJ. Gao) independently. They were blinded against patients’ demographics and previous examination findings. At the start of the study, two researchers had approximately 4 years of experience in assessing lower abdominal and pelvic floor magnetic resonance images (500–800). In addition, two researchers confirmed the diagnostic criteria before the study and participated in consensus meetings in which 25 magnetic resonance images were discussed.

**Figure 3:**
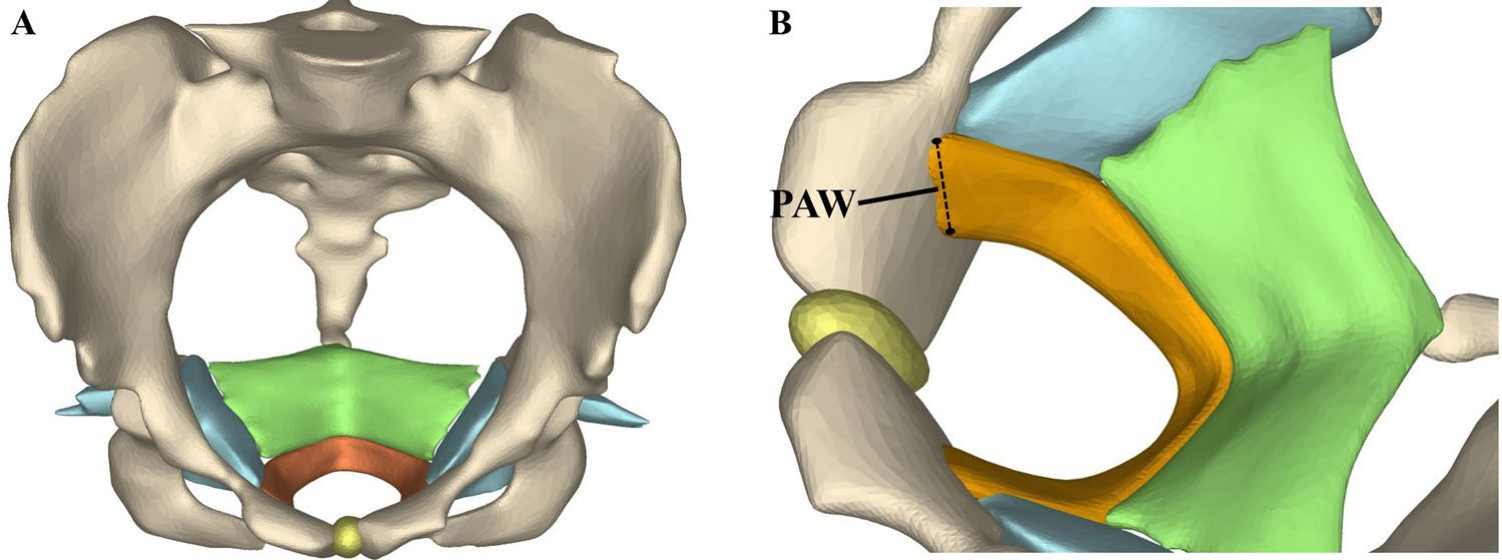
(**A**) The three-dimensional magnetic-resonance based model of pelvic floor. (**B**) The measurement of the puborectalis attachment width (PAW). Showing structures in color. The puborectalis in orange, the iliococcygeus in green, the internal obturator muscle in blue, the pelvic bone in light yellow and the pubic symphysis in dark yellow.

The magnetic resonance images were then imported into Mimics 17.0 (Materialise Group, Leuven, Belgium). On the basis of the anatomy, puborectalis, iliococcygeus and pubic ramus were delineated on consecutive axial scans, which could be reconstructed in 3-dimensional rendering^16,22^. The advanced imaging processing tools were applied, such as smoothing and wrapping^22^ (Fig. 3A). Then the PAW was measured on three-dimensional MRI-based models (3D-MR-model) three times (Fig. 3B). The averaged values were compared with the figures obtained by 2D-US^23^.

### Statistical analysis

The statistical analysis was undertaken by SPSS 17.0 for Windows (SPSS Chicago, IL, USA). The agreement between the methods and observers was assessed by Cohen’s kappa. The value of kappa less than 0.20 indicates poor, 0.21–0.40 fair, 0.41–0.60 moderate, 0.61–0.80 good, and 0.81–1.00 excellent agreement. The parameters were shown as the mean positive and negative standard deviation (X ± s). The agreement between measuring methods was evaluated by Pearson’s correlation. The Pearson’s coefficient less than 0.20 indicates poor, 0.21–0.40 fair, 0.41–0.60 moderate, 0.61–0.80 good, and 0.81–1.00 excellent agreement. A value of P < 0.05 was considered statistically.

### Statement of ethics

This study was approved by the Ethics Committee affiliated with Fujian Provincial Hospital. All procedures performed in the study involving human participants were in accordance with the 1964 Helsinki declaration and its later amendments. All participants signed the informed consent.
